# Treating open tibular fracture (Type gustillo IIIA) with medullary nail assisted by cortex screws: A case report

**DOI:** 10.1097/MD.0000000000033747

**Published:** 2023-05-12

**Authors:** Yuchen Jiang, Jiaqian Zhou, Bingwang Tang, Xingfei Zhu

**Affiliations:** a Shibei Hospital, Shanghai, China.

**Keywords:** cortex screw, Gustillo IIIA open fracture, intramedullay nail, open fracture, secondary fixtator replacement, tibial fracture

## Abstract

Open fractures of the tibia are mostly caused by high-energy injuries, accompanied by severe soft tissue injuries. Staged operations are reported performing better prognosis in these cases.

**Case summary:**

The author reports a case of open comminuted fracture of the left tibia (GustilloIIIA) and discusses its clinical features and treatment experience with the literature. The patient was admitted to our hospital by an ambulance for half an hour due to pain in her left leg caused by a car collision on an electric bike. During hospitalization, external fixation and an intramedullary nail were sequential used.

**Conclusion:**

The case report suggests that cortex screws are likely benefit the prognosis of severe open tibial fracture.

## 1. Introduction

Open tibial fractures are mostly cause by high energy accident, accompanying severe soft tissue injury.^[1–5]^ Treatment of this kind of fractures often lead to wound infection, bone exposure, nonunion and other complications, which brings great difficulty to treatments. Surgical debridement and internal fixation are often used in treatment of this kind of fractures, and after that, external fixation, intramedullary nail or inner fixation plate should be placed. Although external fixators have great effect in early treatment, but if it is used as terminal fixation, problems like infection, external fixator loosening, inconvenient nursery may emerge.^[6–8]^ This report use cortex screws to avoid such uncertainty.

## 2. Medical records

**Figure 1.**
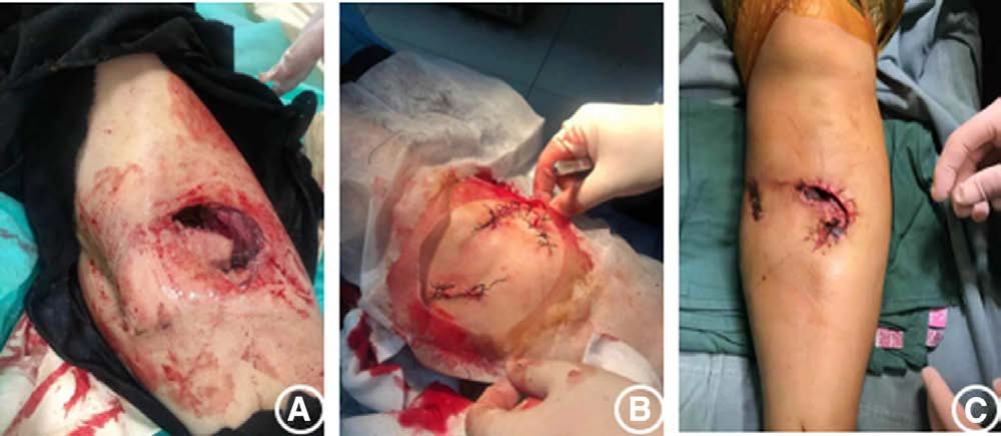
(A) When admitted. (B) After surgical debridement. (C) Before external fixation.

**Figure 2.**
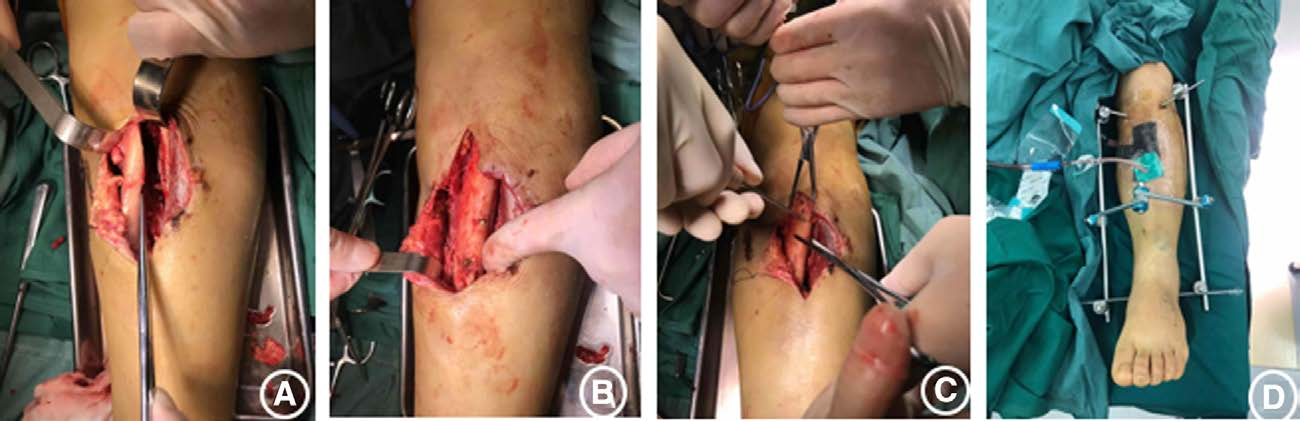
(A–C) Fracture reduction. (D) External fixation.

**Figure 3.**
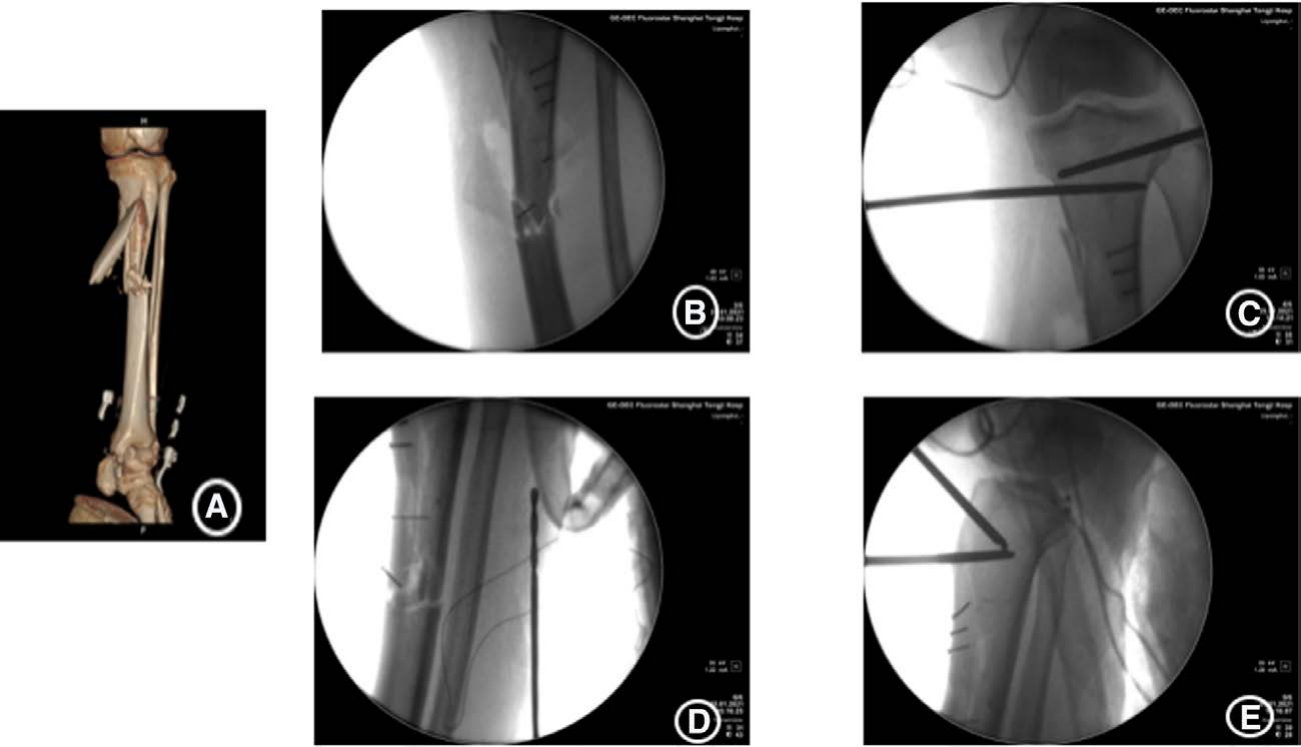
(A) 3-D CT of the fracture. (B–E) C-arm image of the fracture reduction. CT = computerized tomography.

**Figure 4.**
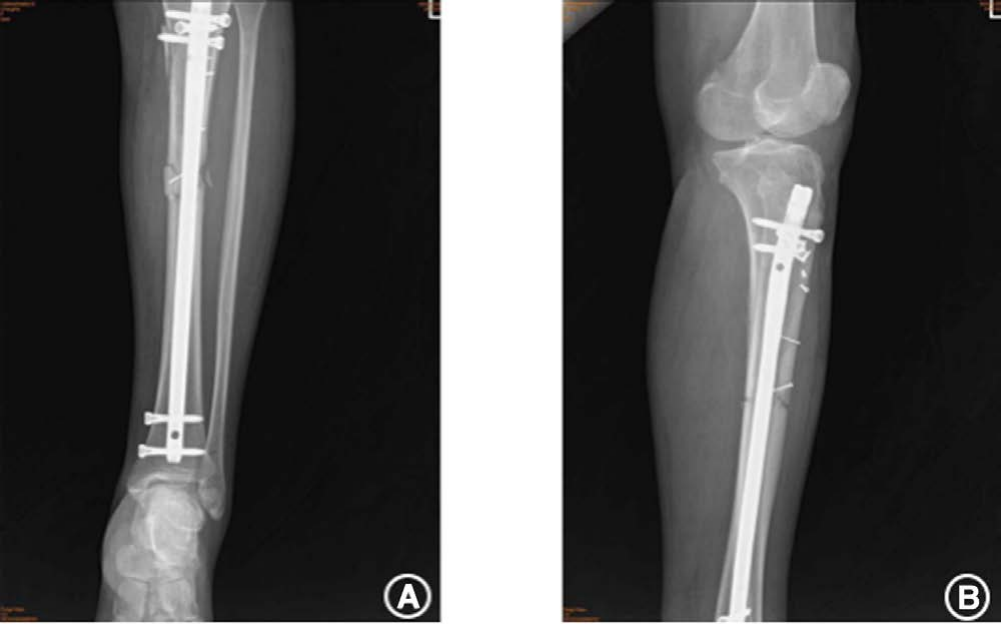
Intramedullary fixation.

**Figure 5.**
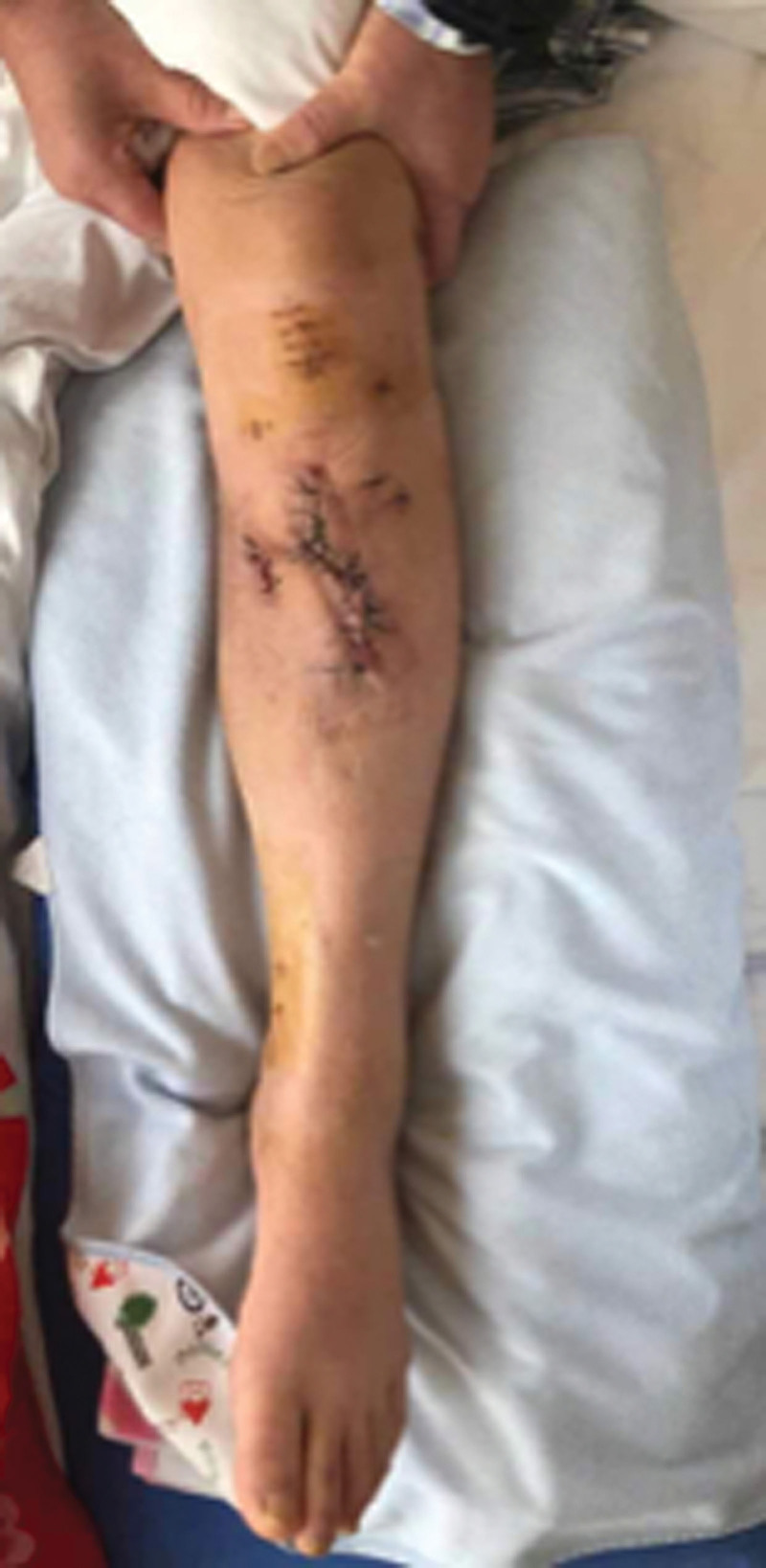
Close wound.

The patient, a 54-year-old woman, with no previous medical illness, was admitted to our hospital by an ambulance for half an hour due to pain in her left leg caused by a car collision on an electric bike. Physical examination after admission showed clear mind and good spirit. An irregular skin fissure about 15 cm in length was seen in the front of the middle part of the left leg, with moderate contamination. The surrounding skin was dark and tender. A 6 cm wound on the medial side of the left leg was seen with a broken bone end. Bone fricatives may be noted. The left lower limb has good blood supply and sensation. The right lower limb is motile, well vascularized, and has good sensation (Fig. 1A). There was no special history. Immediately complete the relevant examination, computerized tomography showed a comminuted fracture of the middle and upper segment of the left tibia, soft tissue swelling of the leg with scattered pneumatosis. The fracture classification: Arbeitsgemeinschaftfür Osteosynthesefragen/Orthopaedic Trauma Association:42B2.1, soft tissue injury classification: GustilloIIIA. The patient received debridement and suture (Fig. 1B), left calcaneus traction, dressing change, anti-infection and detumescence treatment after being admitted to the ward. One week later, skin border necrosis at the debridement and suture was found (Fig. 1C). Blood routine, procalcitonin and C-reactive protein were reviewed on January 16, 2021: white blood cell (WBC) 5.65*10^9/L, C-reactive protein 13.06 mg/L, procalcitonin 0.027 ng/mL. So on January 25, 2021, under general anesthesia, the left tibial open comminuted fracture was treated with internal cortical screw fixation and vaccum sealing drainage (VSD) of the left leg external fixator. During the operation, the fracture end was fixed with a 2.0 mm cortical screw (Figs. 2–3). After the operation, anti-infection and prevention of deep vein thrombosis were continued. On January 30, 2021, blood routine and procalcitonin: WBC 4.98*10, C-reactive protein 10.82 mg/L and procalcitonin 0.020 ng/mL continued to undergo VSD replacement under local anesthesia on February 01, 2021. Necrotic skin was removed during the operation, and some bone was still exposed. VSD aspiration was continued. Blood routine, procalcitonin, erythrocyte sedimentation rate, CRP were reviewed on February 5, 2021: WBC 6.00*10 9/L and C-reactive protein 2.8 mg/L. Procalcitonin was 0.039 ng/mL and erythrocyte sedimentation rate was 38 mm/h. On February 09, 2021, the left tibial fracture was treated with intramedullary nail fixation and external fixation and stent removal. The skin necrosis of the wound was significantly better than before. Some skin incisions were closed, and some bone was still exposed. VSD drainage was continued (Fig. 4A–B). On February 19, 2021, debridement and VSD drainage were performed again under local anesthesia. No obvious skin necrosis and no bone exposure were found. On February 23, 2021, blood routine, procalcitonin, erythrocyte sedimentation rate, CRP: WBC 5.54*10^9/L, C-reactive protein 0.51 mg/L, procalcitonin 0.028 ng/mL were reviewed. The VSD was removed in the ward on February 26, 2021, and the wound was closed (Fig. 5). At present, the patient recovered well, satisfied and was discharged.

## 3. Discussion

Open tibial shaft fractures are common injuries, of which about 20% are Gustillo type III fractures. These severe fractures and soft tissue injuries can cause complications such as infection, fracture nonunion, and skin necrosis, making their treatment difficult. This type of fracture is generally treated in stages, with primary soft tissue debridement and temporary fixation. Later fixation was performed with intramedullary nailing or plate depending on the soft tissue condition. The advantage of intramedullary nail fixation is that the periosteum and soft tissues do not need to be dissected too much, and the central fixation strength is high. It is a common treatment for tibial shaft open fractures. The method of plate internal fixation requires complete exposure of the fracture end, and full dissection of the soft tissue and periosteum, which has a great impact on the soft tissue and seriously affects the blood supply. Temporary fixation of open comminuted fractures requires careful preoperative planning to ensure fixation stability and soft tissue coverage. The traditional practice favors the use of external fixators, whose pins are far away from the injured area and do not affect the soft tissues. However, for tibial comminuted fractures, the stability of fracture fixation cannot be guaranteed by using external fixator alone for one-stage fixation. Some studies have proposed to use external fixator combined with small plate for one-stage fixation without taking out the small plate during the second-stage fixation, so as to ensure the early reduction of fracture and avoid limb deformity caused by long waiting time for final fixation.

For open comminuted tibial shaft fractures, using intramedullary nail as secondary internal fixation has the advantages of minimally invasive, effective stabilization of the long bone for central fixation and allowing early weight-bearing after surgery. The present study suggests that this is the best treatment for an open GustilloIIIA fracture of the tibia. Several previous studies have proposed various methods to maintain fracture reduction during intramedullary nail insertion, including the use of percutaneous traction, blocking screws, or external fixators. However, the above-mentioned measures to maintain the fracture generally need to be removed after the insertion of the intramedullary nail. For comminuted tibial shaft fractures, secondary fixation with intramedullary nailing alone is likely to result in insufficient fixation strength at the fracture end, deformation and displacement of the fracture end, and delayed or nonunion of the fracture.

Assisted fixation of the broken end of a comminuted tibial fracture is a commonly used technique to achieve intramedullary fixation after intramedullary nailing while maintaining fracture reduction. 1/3 tubular plates, reconstruction plates and compression plates have been studied and proposed to be used for auxiliary fixation. Although plates were used for auxiliary fixation in open tibial fractures, Dunbar et al removed these auxiliary fixation plates after intramedullary nailing was inserted, while Archdeacon Wyrick retained the plate as an auxiliary fixation to avoid fracture end deformation.

In theory, there is a risk of infection and nonunion with the retention of the auxiliary plate due to the need for additional periosteal stripping or biofilm formation around the implant during application. Based on this, the author proposes that using cortical screws instead of plates to fix the fracture end is helpful to avoid the risk of using plates for auxiliary fixation, without excessive periosteum dissection and separation of soft tissue, and can protect the blood supply of soft tissue to the maximum extent and enhance the stability of fracture end. In the treatment of this case, 2.0 mm cortical screws were used to fix the fracture end in the first stage, while external fixator was used to maintain the force line of lower limbs, which gained valuable time for the second stage of intramedullary nail fixation and achieved good clinical results. The cortical screw fixation method described in this article has certain advantages over previous treatments. Screw fixation of fractures is a technique that is familiar to all orthopedists. Orthopedists and operating room staff are familiar with the operation and equipment. Even in the treatment of large-scale wounded patients, it can be carried out efficiently without complicated learning curve. Moreover, compared with plate fixation, cortical screw fixation does not require excessive dissection of periosteum and soft tissue, and has less damage to soft tissue. The later period had no effect on the replacement of intramedullary nail fixation. In addition, compared with temporary fixation using steel plates, the cost is greatly reduced and it is a cheaper option.

To sum up, the author believes that this kind of cortical screw combined with intramedullary nail has certain advantages in the fixation of open comminuted GustlioIIIA fracture of the left tibia. It has little damage to soft tissue, does not need excessive periosteum peeling, and is beneficial to fracture healing. It also avoids the risks of infection, delayed bone healing and nonunion of plate fixation. At the same time, due to the displacement of the fracture block, sufficient fixation can be given to prevent the fracture block from deforming. At the same time, the author also proposed that this method should be applied to the cortical area of tibia with sufficient thickness of cortex, preferably at the tibial ridge. The diameter of the screw was 2.0mm. It can be used in combination with external fixator during debridement primary fixation, which not only ensures adequate fracture reduction, but also leaves enough space for later use of intramedullary nailing.

## Author contributions

**Data curation:** Yuchen Jiang.

Formal analysis: Yuchen Jiang.

Investigation: Yuchen Jiang, Xingfei Zhu.

Methodology: Yuchen Jiang, Jiaqian Zhou, Xingfei Zhu.

Resources: Jiaqian Zhou.

Software: Yuchen Jiang.

Supervision: Yuchen Jiang, Jiaqian Zhou.

Validation: Xingfei Zhu.

Visualization: Yuchen Jiang, Xingfei Zhu.

Writing – original draft: Yuchen Jiang.

Writing – review & editing: Yuchen Jiang, Bingwang Tang.
